# Transplant Trial Watch

**DOI:** 10.3389/ti.2025.14654

**Published:** 2025-04-15

**Authors:** Simon R. Knight, John Fallon

**Affiliations:** ^1^ Nuffield Department of Surgical Sciences, Centre for Evidence in Transplantation, University of Oxford, Oxford, United Kingdom; ^2^ Oxford Transplant Centre, Churchill Hospital, Oxford, United Kingdom

**Keywords:** randomised controlled trial, liver transplantation, kidney transplantation, antibody mediated rejection, immunosupression

To keep the transplantation community informed about recently published level 1 evidence in organ transplantation ESOT and the Centre for Evidence in Transplantation have developed the Transplant Trial Watch. The Transplant Trial Watch is a monthly overview of 10 new randomised controlled trials (RCTs) and systematic reviews. This page of Transplant International offers commentaries on methodological issues and clinical implications on two articles of particular interest from the CET Transplant Trial Watch monthly selection. For all high-quality evidence in solid organ transplantation, visit the Transplant Library: www.transplantlibrary.com.

**Randomised Controlled Trial 1**

Randomized Trial Investigating the Utility of a Liver Tissue Transcriptional Biomarker in Identifying Adult Liver Transplant Recipients Not Requiring Maintenance Immunosuppression.

*by Vionnet, J., et al. American Journal of Transplantation 2024 [record in progress].*

## Aims

They aim to assess whether a previously derived 5-gene liver tissue transcriptional biomarker accurately identifies stable adult liver transplant (LT) recipients who can safely discontinue maintenance immunosuppression (IS) without rejection—referred to as operational tolerance.

## Interventions

Divided into two arms: Arm A (Non-biomarker-based): All participants underwent a gradual IS weaning protocol, regardless of biomarker results. Arm B (Biomarker-based): Participants first underwent a liver biopsy, which was tested using the 5-gene “tolerance” biomarker: Arm B+ (biomarker-positive): Proceeded to the same IS weaning protocol as in arm A. Arm B– (biomarker-negative): Continued baseline IS with no weaning.

## Participants

116 adult, stable LT recipients, at least 3 years post-transplant if age >50 or ≥6 years if age ≤50, with normal allograft function, no active viral disease/autoimmune condition, no recent rejection. Arm A: 58 patients and Arm B: 58 patients (24 biomarker-positive, 34 biomarker-negative).

## Outcomes

1. Primary Outcome: Operational tolerance at 1-year post–IS withdrawal, defined by (i) successful IS discontinuation for ≥12 months, (ii) normal liver function, and (iii) absence of rejection or inflammation on protocol biopsy. 2. Secondary Outcomes: Rate, severity, and timing of acute rejection; histologic changes on protocol biopsies; formation of donor-specific antibodies; biomarker performance (sensitivity, specificity, predictive values); immune characterisation (circulating T- and B-cell subsets, RNA-seq of liver tissue, immunohistochemistry measures of immune synapses).

## Follow-Up

At least 1 year after cessation of IS (with a 1-year protocol biopsy). Some participants had extended follow-up to 2 years post–IS withdrawal for additional histologic assessments.

## CET Conclusion

*by John Fallon*

The authors present the LIFT trial, a phase IV, open-label, prospective, multi-centre, randomised controlled trial assessing transcriptional biomarkers for operational tolerance in liver transplantation. Stable livers transplant patients were randomised into one of Arm A, where IS was progressively weened regardless of biomarker status or Arm B, in which biomarker positive patients were offered IS withdrawal and those who were biomarker negative remained on baseline IS. Over all the biomarker, the 5-gene liver transcriptional test, failed to predict who would tolerate IS withdrawal (sensitivity 54%, specificity 42%, positive predictive value ∼16%). They found a low prevalence of operational tolerance, at only 16% (13/80) of patients fully weaned off IS at 1-year post-withdrawal met histologic criteria for operational tolerance. They found that indicators of tolerance to be longer time since transplant and older recipient age, and circulating exhausted/senescent CD8^+^ T cells. Whereas *De novo* donor-specific antibodies were strongly associated with failure of IS withdrawal. The trial found comparable rates of true operational tolerance (∼16%) with the OPTIMAL trial, which had a near identical protocol. They ended recruitment early when interim analysis indicated the biomarker’s positive predictive value was unlikely to meet pre-specified criteria, while ethically justified and common in futility analyses, it reduced the final sample size and power, especially for the low-prevalence outcome of operational tolerance. Operational tolerance was adjudicated mainly at 1 year post-IS withdrawal with a follow-up biopsy. Some participants with mild inflammation at 1 year remained off immunosuppression and eventually stabilized on subsequent histologic checks, meaning the “final” tolerance outcomes might not be fully captured in a single 12-month time point, which given the low overall event rate, could be relevant. The minor methodological concerns revolve around the open-label design, the premature closure of recruitment, and the inherent difficulty of studying an event that is both rare and histologically stringent such as operational tolerance. These factors constrain the ultimate power and precision in estimating the biomarker’s predictive value. Nevertheless, the study’s careful design, protocol harmonization with the OPTIMAL trial, and robust immunologic/histologic analyses make a strong case for the likely negative utility of the 5-gene liver tissue transcriptional biomarker.

## Jadad Score

2.

## Data Analysis

Per protocol analysis.

## Allocation Concealment

Yes.

## Trial Registration

ClinicalTrials.gov - NCT024989977; ISRCTN - 47808000; EudraCT - 2014-004557-14.

## Funding Source

Non-industry funded.

**Randomised Controlled Trial 2**

Donor-Derived Cell-Free DNA Monitoring for Early Diagnosis of Antibody-Mediated Rejection After Kidney Transplantation: A Randomized Trial.

*by Akifova, A., et al. Nephrology Dialysis Transplantation 2024 [record in progress].*

## Aims

This study aimed to determine if monitoring donor-derived cell-free DNA (dd-cfDNA) could lead to early diagnosis of antibody-mediated rejection (AMR) in kidney transplant recipients.

## Interventions

Participants were randomly assigned to either dd-cfDNA-guided kidney allograft biopsy or clinician-guided biopsy.

## Participants

40 adult kidney transplant recipients >180 days following kidney transplantation, with prevalent dnDSA and an estimated glomerular filtration rate (eGFR) ≥20 mL/min/1.73 m^2^.

## Outcomes

The primary outcome was the time from study inclusion to diagnosis of active AMR or chronic active AMR. Secondary outcomes were time from first dnDSA occurrence to the diagnosis of AMR and diagnostic test metrics.

## Follow-Up

24 months after baseline.

## CET Conclusion

*by Simon Knight*

This small, single-centre RCT investigated the potential role of donor derived cell-free DNA (cfDNA) monitoring in kidney transplant recipients with *de novo* DSA. Patients were randomised to routine cfDNA monitoring with biopsy at a threshold of >50 copies/mL, *versus* biopsy for clinical indication. The primary endpoint of time to diagnosis of antibody mediated rejection (AMR) was significantly shorter in the cfDNA group (2.8 months vs. 14.5 months). There are very few prospective RCTs of biomarkers for post-transplant monitoring, and so the authors should be congratulated. It should be noted that the study is open-label and there is a risk of measurement bias, as biopsies in the control group were at the discretion of the clinical team. Whilst the time to diagnosis was shorter in the cfDNA group, the study is too small to demonstrate whether this improves clinical outcomes through earlier treatment, and so is unable to truly assess the benefits of routine monitoring.

## Jadad Score

2.

## Data Analysis

Per protocol analysis.

## Allocation Concealment

Yes.

## Trial Registration

ClinicalTrials.gov - NCT04897438.

## Funding Source

Industry funded.

## Clinical Impact Summary

*by Simon Knight*

Antibody-mediated rejection (AMR) in renal transplant recipients is associated with poor outcomes and graft loss, and is often more resistant to treatment than cellular rejection [1]. Mainstay of diagnosis is biopsy in response to deranged graft function, along with detection of donor specific antibodies (DSA). Waiting for biochemical evidence of graft dysfunction means that diagnosis is often late. Biomarkers that can detect AMR in the earlier stages may allow earlier diagnosis and improve response to treatment. Some centres monitor for *de novo* DSA (dnDSA) routinely, but this strategy is expensive, there is no agreement on the frequency of monitoring for optimal detection, and not all dnDSA lead to rejection [2].

Alternative blood- or urine-based biomarkers may afford more sensitive, non-invasive tools for earlier detection of AMR. One promising candidate is detection of donor-derived cell-free DNA (dd-cfDNA). Graft injury results in release of donor DNA into the recipient circulation, which can be differentiated from recipient cfDNA resulting in a sensitive marker of graft injury. A number of studies have demonstrated strong performance of dd-cfDNA in detection of graft injury resulting from AMR, with high negative predictive values [3]. Despite this promise, there are very few prospective studies examining the impact of biomarker-based monitoring on clinical outcomes.

In a recent study published in Nephrology, Dialysis, Transplantation, Akifova and colleagues examined the role of targeted dd-cfDNA monitoring in kidney transplant recipients with dnDSA [4]. 40 recipients with dnDSA were randomised to 3-monthly dd-cfDNA monitoring, or standard of care, with biopsies performed in the study group where elevated cfDNA levels were detected. They demonstrated that dd-cfDNA monitoring resulted in earlier diagnosis of AMR in the study group (2.8 months vs. 14.5 months). dd-cfDNA monitoring had 77% positive predictive value and 85% negative predictive value for AMR.

There are some limitations to the study. In the control group, decision to biopsy was at the discretion of the clinician caring for the patient, which in an open-label study may result in measurement bias. There were some delays in biopsy in response to elevated cfDNA due to concurrent illness or logistical constraints, which may have reduced the true effect. Whilst rejection was detected earlier, there is no evidence that this earlier diagnosis results in improved clinical outcomes, and the lack of reliable, effective treatment for AMR may limit the value of monitoring at present. A larger sample would be required to assess the true clinical benefit of routine monitoring.

Nonetheless, this is one of the few prospective studies of prospective biomarker monitoring in the literature, showing promise for earlier diagnosis that may impact outcomes in the face of emerging new therapies for AMR.

### Clinical Impact

3/5.
